# A Prospective, Randomized Comparison of Intravitreal Triamcinolone Acetonide Versus Intravitreal Bevacizumab (Avastin) in Diffuse Diabetic Macular Edema

**DOI:** 10.4103/0974-9233.65496

**Published:** 2010

**Authors:** Maha M. Shahin, Rasheed S. El-Lakkany

**Affiliations:** Department of Ophthalmology, Faculty of Medicine, Mansoura University, Egypt

**Keywords:** Bevacizumab, Diabetic Macular Edema, Intravitreal Triamcinolone, Retina, Vascular Endothelial Growth Factor

## Abstract

**Purpose:**

To compare the functional and anatomical outcomes following intravitreal triamcinolone acetonide vs. intravitreal bevacizumab (Avastin) treatment for diffuse diabetic macular edema.

**Materials and Methods:**

In this prospective, randomized study, subjects were divided into two groups: 24 eyes that received intravitreal injection of 4 mg/0.1 mL triamcinolone acetonide (IVTA group) and 24 eyes received intravitreal injection of 1.25 mg/0.05 mL bevacizumab (IVB group). Changes in best corrected visual acuity (BCVA), intraocular pressure (IOP), baseline fluorescein angiography and optical coherence tomography measurements were evaluated in both groups. Follow-up visits out to three months from baseline are reported.

**Results:**

One month after treatment, baseline foveal thickness decreased from 452 µ to 299 µ in the IVTA group and from 292 µ to 270 µ in the IVB group. BCVA increased by two or more lines in 58.3% of eyes in the IVTA group and there was no similar improvement in the IVB group. In the IVTA group, a transient increase in IOP (27–43 mmHg) occurred in four cases (16.7%), which was successfully controlled with topical medications. There were no complications in the IVB group.

**Conclusion:**

Short term outcomes indicate that intravitreal injection of bevacizumab was not associated with surgical complications compared to triamcinolone acetonide. Triamcinolone acetonide appears to be more effective treatment for diabetic macular edema than bevacizumab.

## INTRODUCTION

Diabetic macular edema (DME) is the major cause of visual impairment worldwide.^1^ Based on the observations of the early treatment diabetic retinopathy study (ETDRS), focal/grid laser photocoagulation is the accepted standard of care for DME. However, only 17% of eyes showed any improvement in visual acuity (VA), and less than 3% of eyes experienced improvement of three or more lines after laser treatment.^2^–^4^ In diffuse DME, the edema resolved in 68–94% of cases and visual acuity stabilized in 61% of cases. However, visual acuity decreased by three or more lines in 24.6% of eyes despite treatment.^5^

Alternate treatments for DME are currently under investigation. For example, macular edema has been successfully reversed by intravitreal injection of varying doses (1 to 21 mg) of triamcinolone acetonide in uveitis, retinal vein occlusion, chronic pseudophakic cystoid macular edema, radiation retinopathy and juxtafoveal telangiectasia.^6^–^12^ The most common risks of intravitreal corticosteroids are mild to moderate elevation of intraocular pressure (IOP) and the development of cataract.^13^,^14^

Although the pathogenesis of DME remains unknown, vascular endothelial growth factor (VEGF) seems to play a role. Elevated levels of VEGF in patients with DME compared to diabetics without maculopathy have been reported.^15^ The upregulation of VEGF is associated with breakdown of the blood-retinal barrier, with increased vascular permeability resulting in retinal edema.^16^ Bevacizumab (Avastin, Genentech Inc, San Francisco, CA, USA), a recombinant human monoclonal antibody directed against VEGF has been used for cancer treatment.^17^ Intravitreal bevacizumab has emerged as a therapeutic strategy for retinal diseases such as age-related macular degeneration and macular edema due to central retinal vein occlusion.^18^,^19^ Hence, it is reasonable to assume that VEGF inhibitors such as bevacizumab will also be applicable in other retinal diseases such as DME. The purpose of this study was to compare functional and anatomic outcomes of intravitreal triamcinolone acetonide and intravitreal bevacizumab in diffuse macular edema.

## MATERIALS AND METHODS

This was a prospective, randomized, study that included 48 eyes of 32 subjects with diffuse macular edema not associated with vitreomacular traction. Diffuse DME was defined as retinal thickening measuring one disc diameter or greater with generalized leakage on fluorescein angiography and concomitant vision decrease. None of the patients included in this study had prior laser therapy.

All subjects underwent an ophthalmic examination that included measurement of best corrected snellen visual acuity (BCVA), optical coherence tomography (OCT) and fundus fluorescein angiography at presentation. Fluorescein angiography was performed with digital images acquired every second upon injection of the dye until filling of retinal veins and acquisition of images of the macula during the late phase. OCT of each eye was performed with six linear scans oriented radially 30° apart and centered on the fovea. Central macular and foveal thicknesses were measured within a 3.45 mm diameter centered on the fovea. The circular map was subdivided into nine quadrants with the middle and the inner diameters at 2.22 mm and 1.00 mm, respectively.

The study cohort was divided into two groups using a randomization schedule. One group of subjects (24 eyes) received a single intravitreal injection of (4 mg in 0.1 mL) triamcinolone acetonide (IVTA group) and another group of patients (24 eyes) received intravitreal injection of (1.25 mg in 0.05 mL) bevacizumab (IVB group). All treatments were performed under sterile conditions. The eyelids were cleansed with 5% betadine and one to two drops of topical anesthetic (benoxinate hydrochloride 0.4%) were delivered to the eye. Triamcinolone acetonide was injected into the vitreous cavity with a 30-gauge needle with a pars plana approach 3.5 mm inferotemporally from the limbus. Subjects were evaluated at one day, one week, one month and three months after intravitreal injection. At one day and one week follow up, visual acuity, intraocular pressure, evidence of infection or uveitis was assessed. At one month and three months after treatment, the same evaluation as one day was conducted in addition to the assessment of potential cataract formation and repeat fluorescein angiography and OCT.

## RESULTS

Twenty females and 12 males comprised the study cohort. The mean age of the cohort was 52.7 years. In the IVTA group at the one month visit, there was an increase in BCVA of more than two lines in 14 eyes (58.3%), a one line improvement in six eyes (25%), no improvement in four eyes (16.7%) and no loss of BCVA. In the IVTA group, the greatest improvement in visual acuity occurred by a week and remained stable three months after treatment. In the IVB group, at the one month visit, there was an increase of baseline BCVA by one line in 12 eyes (50%), there was no improvement in nine eyes (37.5%), while three eyes (12.5%) lost BCVA by one line or more [Table 1]. In the IVB group the greatest improvement in visual acuity occurred at a week and the remained stable for one month. After the one month visit, visual acuity decreased in the IVB group.

**Table 1:** Change in best corrected visual acuity of 48 eyes that underwent intravitreal triamcinolone acetonide therapy or intravitreal bevacizumab therapy

	No.	↓BCVA		No change		1 line improvement		≥2 lines improvement	
		No.	%	No.	%	No.	%	No.	%
IVTA group	24	-	-	4	16.7	6	25	14	58.3
IVB group	24	3	12.5	9	37.5	12	50	-	-

No.: number of eyes, BCVA: Best Corrected Visual Acuity, ↓BCVA: One line or more decrease in Best Corrected Visual Acuity, IVTA group: Eyes that received intravitreal triamcinolone acetonide, IVB group: Eyes that received intravitreal bevacizumab

**Figure 1:**
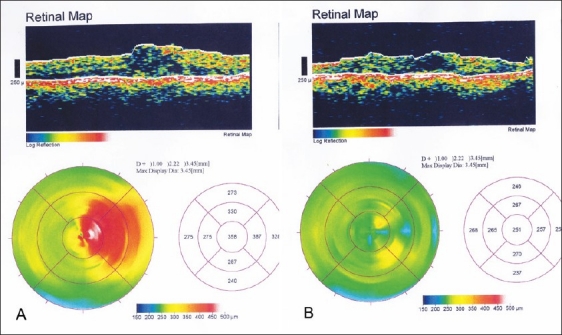
Optical coherence tomography of a 59-year-old male diabetic with cystoid macular edema A) before and B) three months after IVTA intravitreal triamcinolone acetonide injection

**Figure 2:**
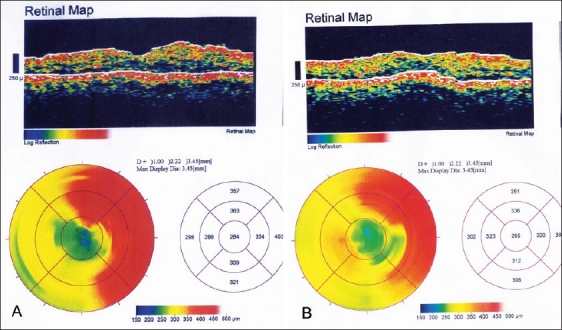
Optical coherence tomography of a 62-year-old female diabetic with diffuse diabetic macular edema A) before and B) three months after intravitreal bevacizumab injection

The mean foveal thickness one month after treatment decreased by 34% in the IVTA group [Figure 1] and by 7.5% in the IVB group [Table 2]. There was an increase in foveal thickness after the one month follow-up visit in the IVB group [Figure 2].

**Table 2:** Foveal thickness before and one month after receiving intravitreal triamcinolone acetonide or intravitreal bevacizumab

	No.	Mean OCT “baseline” (µm)	Mean OCT “postop” (µm)	Percent decrease in thickness (%)
IVTA group	24	452	299	34
IVB group	24	292	270	7.5

No.: number, OCT: Optical coherence tomography, Postop: Postoperative, IVTA group: Eyes that received intravitreal triamcinolone acetonide, IVB group: Eyes that received intravitreal bevacizumab

There were no complications in the IVB group for the entire duration of the study. In the IVTA group, a transient increase in the IOP (27–43 mmHg) occurred in four eyes (16.7%). All four cases were successfully controlled with topical medications by the end of the study. Visually significant cataract did not occur in both groups during this study.

## DISCUSSION

A number of studies have reported the use of triamcinolone to improve visual acuity and/or reduce macular thickness due to macular edema.^6^–^12^ Consistent with previous reports, we found a significant reduction of foveal thickness and improved visual acuity in subjects who were administered triamcinolone acetonide.^6^,^20^

The mechanism that reduces macular edema due to intravitreal administration of triamcinolone acetonide has yet to be determined. However, the rationale for this therapy is based on the ability to inhibit the arachidonic acid pathway, of which prostaglandin is a product. Corticosteroids may also downregulate the production of VEGF. Additionally, a reduction of the breakdown of the blood-retinal barrier due to triamcinolone acetonide has been reported.^21^

The main side effects of intravitreal triamcinolone observed in our study was an increase in IOP. This increase has been previously reported.^13^–^14^ The secondary ocular hypertension was successfully lowered using topical medications without glaucomatous optic nerve damage.

In this study we found relatively poor resolution of macular edema to intravitreal bevacizumab. The most likely reason for the muted response to bevacizumab is that diabetic macular edema is not entirely mediated by VEGF. In contrast intravitreal triamcinolone has a number of mechanisms of action that make it effective.

Animal studies have reported the reversal and prevention of retinal neovascularization in rabbits with intravitreal bevacizumab.^22^ However, bevacizumab did not entirely prevent or reverse vascular dilatation and tortuosity.^22^ The authors suggested that the amount of VEGF required for initiation of neovascularization may be lower than that required for the breakdown of the blood-retinal barrier.^22^ These findings are consistent with the poor therapeutic response to intravitreal bevacizumab in cases of macular edema that we report here. There were no complications to intravitreal injection of bevacizumab such as inflammation, increased IOP, retinal tears, or detachment.

The improvement in visual acuity remained stable for three months in the IVTA group and one month in the IVB. In the IVB group, visual acuity and foveal thickness deteriorated after one month. These observations are consistent with previous reports suggesting that measurable concentrations of triamcinolone would be present for approximately three months after a single 4 mg intravitreal injection of triamcinolone acetonide (as administered in our study) in the absence of a vitrectomy.^23^ Pharmacokinetic data suggest a single dose of intravitreal injection of 1.25 mg/0.05 mL bevacizumab is expected to be effective for 6–7 weeks.^24^ The limitations of this study are the short duration of follow up and the limited number of patients. However, we tried to partially mitigate these drawbacks by incorporating a prospective study design and randomization.

In conclusion, the data from our study indicate that intravitreal injection of 4 mg of triamcinolone acetonide may improve visual outcomes in patients with diffuse diabetic macular edema without major complications. Intravitreal triamcinolone acetonide in diffuse diabetic macular edema provided better visual and anatomical outcomes compared to intravitreal bevacizumab.
